# Supplementary material for: Zika and dengue but not chikungunya are associated with Guillain-Barré syndrome in Mexico: A case-control study
Source: PLoS Negl Trop Dis. 2020 Dec 17;14(12):e0008032. doi: 10.1371/journal.pntd.0008032 (PMC7775118; doi:10.1371/journal.pntd.0008032)
Supplement: S1 STROBE Statement Checklist — (DOCX) [file pntd.0008032.s001.docx]

|  | **Section & Topic** | **Item**  **No** | **Recommendations** | **Section´s paragraph** |
| --- | --- | --- | --- | --- |
|  | **Title and abstract** | **1** |  |  |
|  |  |  | (a) Indicate the study’s design with a commonly used term in the title or the abstract | Title paragraph |
|  |  |  | (b) Provide in the abstract an informative and balanced summary of what was done and what was found | Abstract paragraphs |
|  | **Introduction** |  |  |  |
|  | Background/rationale | **2** | Explain the scientific background and rationale for the investigation being reported | 1-4 |
|  | Objectives | **3** | State specific objectives, including any pre-specified hypotheses | 4 |
|  | **Methods** |  |  |  |
|  | Study design | **4** | Present key elements of study design early in the paper | 2 |
|  | Setting | **5** | Describe the setting, locations, and relevant dates, including periods of recruitment, exposure, follow-up, and data collection | 3-7 |
|  | Participants | **6** | (a) Give the eligibility criteria, and the sources and methods of case ascertainment and control selection. Give the rationale for the choice of cases and control | 3-7 |
|  |  |  | (b) For matched studies, give matching criteria and the number of controls per case | 4 |
|  | Variables | **7** | Clearly define all outcomes, exposures, predictors, potential confounders, and effect modifiers. Give diagnostic criteria, if applicable | 3-10 |
|  | Data sources/ measurement | **8*** | For each variable of interest, give sources of data and details of methods of assessment (measurement). Describe comparability of assessment methods if there is more than one group | 11-18 |
|  | Bias | **9** | Describe any efforts to address potential sources of bias | This item was not considered |
|  | Study size | **10** | Explain how the study size was arrived at | 19 |
|  | Quantitative variables | **11** | Explain how quantitative variables were handled in the analyses. If applicable, describe which groupings were chosen and why | Does not apply |
|  | Statistical methods | **12** | (a) Describe all statistical methods, including those used to control for confounding | 19 |
|  |  |  | (b) Describe any methods used to examine subgroups and interactions | It was not done |
|  |  |  | (c) Explain how missing data were addressed | Does not apply |
|  |  |  | (d) If applicable, explain how matching of cases and controls was addressed | Does not apply |
|  |  |  | (e) Describe any sensitivity analyses | Does not apply |
|  | **Results** |  |  |  |
|  | Participants | **13*** | (a) Report numbers of individuals at each stage of study—eg numbers potentially eligible, examined for eligibility, confirmed eligible, included in the study, completing follow-up, and analysed | 1-6. Tables 1 and 2 |
|  |  |  | (b) Give reasons for non-participation at each stage | Does not apply |
|  |  |  | (c) Consider use of a flow diagram | This is not necessary |
|  | Descriptive data | **14*** | (a) Give characteristics of study participants (eg demographic, clinical, social) and information on exposures and potential confounders | 1-12  Table 1-5 |
|  |  |  | (b) Indicate number of participants with missing data for each variable of interest | Does not apply |
|  | Outcome data | **15*** | Report numbers in each exposure category, or summary measures of exposure | 5-6 |
|  | Main results | **16** | (a) Give unadjusted estimates and, if applicable, confounder-adjusted estimates and their precision (eg, 95% confidence interval). Make clear which confounders were adjusted for and why they were included | 5, 6, 8, 9 |
|  |  |  | (b) Report category boundaries when continuous variables were categorized | It was not done |
|  |  |  | (c) If relevant, consider translating estimates of relative risk into absolute risk for a meaningful time period | It was not done |
|  | Other analyses | **17** | Report other analyses done—eg analyses of subgroups and interactions, and sensitivity analyses | It was not done |
| **Discussion** | | | | |
|  | Key results | **18** | Summarise key results with reference to study objectives | 1 |
|  | Limitations | **19** | Discuss limitations of the study, taking into account sources of potential bias or imprecision. Discuss both direction and magnitude of any potential bias | 14 |
|  | Interpretation | **20** | Give a cautious overall interpretation of results considering objectives, limitations, multiplicity of analyses, results from similar studies, and other relevant evidence | 2-12 |
|  | Generalisability | **21** | Discuss the generalisability (external validity) of the study results | 13 |
| **Other information** | | | | |
|  |  | **22** | Give the source of funding and the role of the funders for the present study and, if applicable, for the original study on which the present article is based | It is not in the document but in the journal system |
|  |  |  |  |  |
|  |  |  |  |  |
