# Supplementary material for: Zika and dengue but not chikungunya are associated with Guillain-Barré syndrome in Mexico: A case-control study
Source: PLoS Negl Trop Dis. 2020 Dec 17;14(12):e0008032. doi: 10.1371/journal.pntd.0008032 (PMC7775118; doi:10.1371/journal.pntd.0008032)
Supplement: Attachment — Submitted filename: Zika-Dengue-GBS-Stata code.docx [file pntd.0008032.s004.docx]

clear

input str3 state case zika match

BC 1 0 1

BC 1 0 2

BC 1 0 3

BC 1 0 4

BC 1 0 5

BC 1 0 6

BC 1 0 7

BC 1 0 8

BC 1 0 9

BC 1 0 10

BC 0 0 1

BC 0 0 2

BC 0 0 3

BC 0 0 4

BC 0 0 5

BC 0 0 6

BC 0 0 7

BC 0 0 8

BC 0 0 9

BC 0 0 10

BC 0 0 1

BC 0 0 2

BC 0 0 3

BC 0 0 4

BC 0 0 5

BC 0 0 6

BC 0 0 7

BC 0 0 8

BC 0 0 9

CA 1 0 11

CA 0 0 11

CA 0 0 11

MO 1 0 12

MO 0 0 12

MO 0 0 12

CH 1 0 13

CH 0 0 13

CH 0 0 13

NA 1 0 14

NA 0 0 14

NA 0 0 14

NU 1 1 15

NU 1 1 16

NU 1 1 17

NU 1 0 18

NU 1 0 19

NU 1 0 20

NU 1 0 21

NU 1 0 22

NU 1 0 23

NU 1 0 24

NU 1 0 25

NU 1 0 26

NU 1 0 27

NU 1 0 28

NU 1 0 29

NU 1 0 30

NU 1 0 31

NU 1 0 32

NU 1 0 33

NU 1 0 34

NU 1 0 35

NU 1 0 36

NU 1 0 37

NU 1 0 38

NU 1 0 39

NU 1 0 40

NU 1 0 41

NU 1 0 42

NU 1 0 43

NU 1 0 44

NU 1 0 45

NU 1 0 46

NU 1 0 47

NU 1 0 48

NU 1 0 49

NU 1 0 50

NU 1 0 51

NU 0 0 15

NU 0 0 16

NU 0 0 17

NU 0 0 18

NU 0 0 19

NU 0 0 20

NU 0 0 21

NU 0 0 22

NU 0 0 23

NU 0 0 24

NU 0 0 25

NU 0 0 26

NU 0 0 27

NU 0 0 28

NU 0 0 29

NU 0 0 30

NU 0 0 31

NU 0 0 32

NU 0 0 33

NU 0 0 34

NU 0 0 35

NU 0 0 36

NU 0 0 37

NU 0 0 38

NU 0 0 39

NU 0 0 40

NU 0 0 41

NU 0 0 42

NU 0 0 43

NU 0 0 44

NU 0 0 45

NU 0 0 46

NU 0 0 47

NU 0 0 48

NU 0 0 49

NU 0 0 50

NU 0 0 51

NU 0 0 15

NU 0 0 16

NU 0 0 17

NU 0 0 18

NU 0 0 19

NU 0 0 20

NU 0 0 21

NU 0 0 22

NU 0 0 23

NU 0 0 24

NU 0 0 25

NU 0 0 26

NU 0 0 27

NU 0 0 28

NU 0 0 29

NU 0 0 30

NU 0 0 31

NU 0 0 32

NU 0 0 33

NU 0 0 34

NU 0 0 35

NU 0 0 36

NU 0 0 37

NU 0 0 38

NU 0 0 39

NU 0 0 40

NU 0 0 41

NU 0 0 42

NU 0 0 43

NU 0 0 44

NU 0 0 45

NU 0 0 46

NU 0 0 47

NU 0 0 48

NU 0 0 49

TA 1 0 51

TA 0 0 51

TA 0 0 51

QU 1 0 52

QU 0 0 52

VE 1 1 53

VE 1 1 54

VE 1 1 55

VE 1 1 56

VE 1 1 57

VE 1 0 58

VE 1 0 59

VE 1 0 60

VE 1 0 61

VE 1 0 62

VE 1 0 63

VE 1 0 64

VE 1 0 65

VE 1 0 66

VE 1 0 67

VE 1 0 68

VE 1 0 69

VE 1 0 70

VE 1 0 71

VE 1 0 72

VE 1 0 73

VE 1 0 74

VE 1 0 75

VE 1 0 76

VE 1 0 77

VE 1 0 78

VE 1 0 79

VE 1 0 80

VE 1 0 81

VE 1 0 82

VE 1 0 83

VE 1 0 84

VE 1 0 85

VE 1 0 86

VE 1 0 87

VE 1 0 88

VE 1 0 89

VE 1 0 90

VE 1 0 91

VE 1 0 92

VE 1 0 93

VE 1 0 94

VE 1 0 95

VE 1 0 96

VE 0 1 53

VE 0 0 54

VE 0 0 55

VE 0 0 56

VE 0 0 57

VE 0 0 58

VE 0 0 59

VE 0 0 60

VE 0 0 61

VE 0 0 62

VE 0 0 63

VE 0 0 64

VE 0 0 65

VE 0 0 66

VE 0 0 67

VE 0 0 68

VE 0 0 69

VE 0 0 70

VE 0 0 71

VE 0 0 72

VE 0 0 73

VE 0 0 74

VE 0 0 75

VE 0 0 76

VE 0 0 77

VE 0 0 78

VE 0 0 79

VE 0 0 80

VE 0 0 81

VE 0 0 82

VE 0 0 83

VE 0 0 84

VE 0 0 85

VE 0 0 86

VE 0 0 87

VE 0 0 88

VE 0 0 89

VE 0 0 90

VE 0 0 91

VE 0 0 92

VE 0 0 93

VE 0 0 94

VE 0 0 95

VE 0 0 96

VE 0 0 53

VE 0 0 54

VE 0 0 55

VE 0 0 56

VE 0 0 57

VE 0 0 58

VE 0 0 59

VE 0 0 60

VE 0 0 61

VE 0 0 62

VE 0 0 63

VE 0 0 64

VE 0 0 65

VE 0 0 66

VE 0 0 67

VE 0 0 68

VE 0 0 69

VE 0 0 70

VE 0 0 71

VE 0 0 72

VE 0 0 73

VE 0 0 74

VE 0 0 75

VE 0 0 76

VE 0 0 77

VE 0 0 78

VE 0 0 79

VE 0 0 80

VE 0 0 81

VE 0 0 82

VE 0 0 83

VE 0 0 84

VE 0 0 85

VE 0 0 86

VE 0 0 87

VE 0 0 88

VE 0 0 89

VE 0 0 90

end

cc case zika

clogit case zika, group(match) or
