# Supplementary material for: Zika and dengue but not chikungunya are associated with Guillain-Barré syndrome in Mexico: A case-control study
Source: PLoS Negl Trop Dis. 2020 Dec 17;14(12):e0008032. doi: 10.1371/journal.pntd.0008032 (PMC7775118; doi:10.1371/journal.pntd.0008032)
Supplement: Attachment — Submitted filename: Reviewers responses & description manuscript changes.docx [file pntd.0008032.s005.docx]

**REVIEWER 1**

**Methods**

**Authors used methods used in several other countries in Latin America and elsewhere to study the association of flaviviruses and GBS. One limitation is the use of IgMs which may not be enough to differentiate Zika and Dengue. In the past, PRNTs have been obtained to fully differentiate immunologic evidence of recent flavivirus infection.**

R = We agree with the reviewer’s comment. The techniques used for this study are those recommended by international organizations, such as the WHO, and national institutions, as the Health Ministry of Mexico, for the epidemiological surveillance of Dengue, chikungunya and Zika. RT-qPCR was considered the adequate choice for classifying acute infection. When RT-PCR results were negative, IgMs were determined. Unfortunately, we did not use PRTs as it was published before because we followed Dirlikov, et al., (2017) recommendations for laboratory evidence of Zika virus infection if they had a positive RT-PCR result in any specimen or anti-Zika immunoglobulin M (IgM) detected in serum.

**Results**

**Increased mortality may be due to the bias towards sicker patients being included in the study.**

R = We agree with the reviewer’s observation. All the cases included in the study were inpatients, for which mild and outpatient cases were not included for the analysis.

Although mortality was not statistically different from ZIKV-/DENV- patients, it was commented upon as an additional factor contributing to the establishment of the higher severity of the disease.

**Conclusions**

**I would tone down a bit the clinical differences between Zika and dengue in light of unavailability of PRNTs. Otherwise it reads well.**

R = We agree with the reviewer’s point of view. Because we do not demonstrate acute or laboratory evidence of DENV infection, we cannot contrast clinical differences with ZIKV, for which this section will not be considered in the new version of the manuscript.

**Editorial and Data Presentation Modifications**

**Line 365: Verify spelling of Dr. Styczynski last name.**

R = This typo has already been corrected in the manuscript.

**Line 370: Please clarify meaning of ...the biological sample was not specific.**

R = Dirlikov et al. (2017), evaluated the acute Zika virus infection as a risk factor for Guillain-Barre syndrome in Puerto Rico. In this study, the authors collected serum, urine and saliva but did not specify the type of sample used for the analysis.

**Line: 454: Verify meaning of sentence. researchers?**

**Researches decided voluntarily to participate in the study. The participation was not mandatory.**

R = In our institution, epidemiologists indirectly depend on the Coordination of Epidemiological Surveillance, for which their participation in the study could have been mandatory. However, at a specific meeting, they were invited to participate in the development of the protocol on a voluntary basis. Unfortunately, only a few participants decided to contribute to this study, so we think this was one of the reasons for the small sample size obtained. Given this clarification, we consider that it is not necessary, so this sentence will be deleted from the new version of the manuscript.

**Summary and General Comments**

**Overall well written paper. Easy to read. Important contribution to the GBS/flavivirus literature.**

**REVIEWER 2**

**Methods**

**The methods section should be expanded and statistical analysis methods further clarified.**

R = We agree with the reviewer. The suggested modifications have been made in the new version of the manuscript.

**Definition of acute cases versus recent infections not clear.**

R = Acute infection by ZIKV, DENV or CHIKV is defined as a positive result to the reverse transcriptase real-time polymerase chain reaction (RT-qPCR) in either serum or urine samples for ZIKV or serum for DENV or CHIKV.

Laboratory evidence of ZIKV, DENV or CHIKV infection is the adequate terminology instead of recent infection, and is defined as a positive RT-qPCR result in serum or urine samples of ZIKV, DENV or CHIKV, or an anti-ZIKV, anti-DENV or anti-CHIKV immunoglobulin M (IgM) detected in serum.

Detailed description of these definitions are included in the new manuscript

**Clarify if there are overlaps**

R = According to the Pan American Health Organization (PAHO) (Tools for the diagnosis and care of patients with suspected arboviral diseases) although cross-reactions may occur during the serological diagnosis of Dengue and Zika, it has been shown that, in primary infections (first infection with a flavivirus), the antibodies of these patients hardly cross-react with other antigenically related viruses. It should be noted that for the kit used for the determination of IgM antibodies against Zika, the manufacturer did not observe cross-reaction for Dengue in 145 tested samples. While the plate reduction neutralization (PRNT) technique offers greater specificity in the detection of neutralizing antibodies (IgG), it was not used in this study.

**Detail detection limits of instruments used for testing (specificity and timing).**

Response:

a) **Molecular biology techniques**

In the case of Zika, the uniplex technique has a detection limit (sensitivity) of 25 copies. There is no cross-reaction with dengue virus-1 (DENV-1), DENV-2, DENV-3, DENV-4, West Nile virus, St. Louis encephalitis virus, yellow fever virus, Powassan virus, Semliki Forest virus, o'nyong-nyong virus, chikungunya virus or Spondweni virus. For Chikungunya, the uniplex technique has a detection limit (sensitivity) of 0.9 copies. It has no cross-reaction with o'nyong-nyong, Ross River, Mayaro, Semliki Forest, Sindbis, Western equine encephalitis, Eastern equine encephalitis, and Venezuelan equine encephalitis subtypes 1AB, 1C, 1D, and 1E. For Dengue, the uniplex technique has a detection limit (sensitivity) of 2.4, 11.7, 1.5, and 2.3 copies for DENV-1 to DENV-4, respectively.

The commercial kit of RT-qPCR (VIASURE) for Zika, Dengue & Chikungunya (Cat. VS-ZDC112L, Viasure, Zaragoza, Spain) has a detection limit (sensitivity) of 10 copies of Dengue, Chikungunya, and Zika per reaction. The kit has no cross-reaction for the following organisms:

**Probes and primers for Zika**

Chikungunya virus strain S27 Petersfield, St Louis Encephalitis virus strain 17D, Dengue 1 virus strain Hawaii, West Nile virus strain H160/99, Dengue 2 virus strain New Guinea C, West Nile virus Heja, Dengue 3 virus strain H87, West Nile virus Ug37, Dengue 4 virus strain H241, Yellow Fever virus strain 17D, and *Plasmodium falciparum*.

**Probes and primers for Dengue**

Chikungunya virus strain S27 Petersfield, St Louis Encephalitis virus strain 17D, Zika virus strain MR 766 (Uganda), West Nile virus strain H160/99, Zika Virus strain 11474/16 (French Polynesia), West Nile virus Heja, Zika Virus strain 11468/16(French Polynesia), West Nile virus Ug37, Zika Virus (African), Yellow Fever virus strain 17D, Zika virus strain PF13/251013-18 (Asian), *Plasmodium falciparum*, *Trypanosoma cruzi*, and Japanese encephalitis.

**Probes and primers for Chikungunya**

Zika virus strain MR 766 (Uganda), Dengue 3 virus strain H87, Zika Virus strain 11474/16 (French Polynesia), Dengue 4 virus strain H241, Zika Virus strain 11468/16(French Polynesia), St Louis Encephalitis virus strain 17D, Zika Virus (African), West Nile virus strain H160/99, Zika virus strain PF13/251013-18 (Asian), West Nile virus Heja, Dengue 1 virus strain Hawaii, West Nile virus Ug37, Dengue 2 virus strain, New Guinea C, Yellow Fever virus strain 17D, *Plasmodium falciparum*, *Trypanosoma cruzi*, and Japanese encephalitis.

**b)	IgM antibody determinations**

For the Anti-Zika Virus ELISA IgM kit (Cat. EI 2668-9601M, Euroimmun AG, Lübeck, Germany), sensitivity is 56%, and specificity is 97%. The kits showed no cross-reaction with Dengue, patients vaccinated against Central European encephalitis virus (TBE), yellow fever, or patients infected with Japanese encephalitis virus or West Nile virus.

For the Panbio Dengue IgM Capture ELISA kit (Cat. 01PE20/01PE21, Panbio Diagnostics, Korea), the serological sensitivity (primary infection) was 85.4–98.9%; serological sensitivity (secondary infection), 46.6–64.7% and serological specificity (negative), 100%. The kit did not show cross-reactivity with Epstein-Barr virus, Influenza A, Influenza B, Antinuclear antibody, Hepatitis A, Leptospirosis, *Salmonella typhi*, or Scrub typhus.

For the CHIKjj Detect IgM ELISA kit (Cat. CHKM-C, InBios International, Inc. USA), sensitivity and specificity are greater than 90%. The kit did not show cross-reactivity against antinuclear antibody, rheumatoid factor, herpes simplex virus, human immunodeficiency virus, Hepatitis C virus, West Nile virus, Dengue virus, Japanese encephalitis virus, St. Louis encephalitis virus and La Crosse encephalitis virus.

**Results**

**Table 2 should be clarified (acute vs recent infection cases)**

R = Table 2 was modified to differentiate acute vs. laboratory evidence of virus infection cases.

**- Results in the text not consistent with those presented in Table**

R = Results were cross-checked and corrected in both the manuscript and the Table.

**- Modifications to Table 5 suggested; an additional Table suggested**

R = We agree with the reviewer's suggestion. The information on Table 5 was modified.

**Conclusions**

**Limitations need to be expanded. Main limitations not considered.**

R = The following are the main limitations of this manuscript:

1.	Sample size. For an epidemiological study, the sample size was scarce, which prevented to identifying more ZIKV, DENV, or CHIKV cases. Therefore, it was difficult to demonstrate the initially formulated association.

2.	Controls were matched by age, gender, and place of residence. However, other factors such as socioeconomic level were not contemplated, although over 75% of the population in Mexico is considered as poor and vulnerable.

3.	The selection of controls with no fever might have generated an overestimation of the exposure (ZIKV and DENV) in cases and an underestimation in controls. This fact should be considered for future studies, although it was recently shown that a study which only included GBS cases without febrile illness in the last 5 days before the onset of GBS was significantly different from controls without fever (Góngora et al.,2020).

4. Although patients and interviewers were unaware if the recruited cases were at risk of prior infection with any of the arboviruses, the interviews to identify previous diseases or symptomatology—of which individuals might be unaware—could be considered as another limitation of the study

5.	Other causes of GBS were not considered, such as *Campylobacter jejuni*, which causes acute diarrheal disease and can be related to more than 50% of GBS cases which in Mexico. Other respiratory viruses also associated with the presence of GBS were not considered as well.

**Editorial and Data Presentation Modifications**

**The Tables could be better formatted' lack of consistency in digits after the decimal, which would make for a clearer read.**

R = Tables were modified as recommended.

**- Some sentences could be improved for clarity (as marked on pdf)**

R = Unfortunately, we do not have the indicated PDF.

**- Copyediting would help the manuscript flow**

R = The manuscript has been revised and copyedited.

**REVIEWER 3**

**Methods
-Is the study design appropriate to address the stated objectives? NO**

R = We consider a case-control design suitable for the study of rare diseases, such as GBS and its association with ZIKV or DENV or CHIKV, despite the methodological problems involved. This type of study provides information that mirrors what could be learned from a cohort study, usually at considerable less cost and time, despite the two main sources of bias: a separate sampling of cases and controls, and a retrospective measurement of predictive variables.

**-Is the population clearly described and appropriate for the hypothesis being tested? NO**

R = We improved the description of the population in the new version of the manuscript.

**-Is the sample size sufficient to ensure adequate power to address the hypothesis being tested? NO**

R = We agree with this observation. Although the calculated sample size to obtain, at least, an odds ratio of 2 was 15 cases, we were only able to obtain 53% of this number.

**-Were correct statistical analysis used to support conclusions? NO**

R = Considering the methodology, we think that the statistical tests used in the study are appropriate to determine the association between positive laboratory tests and the development of GBS, as well as the analysis of clinical features. More details are described ahead. From a methodological point of view, the pairing was decided by age (±5 years), gender, and place of residence to reduce as much confusion as possible but not for conducting special statistical tests.

--------------------
**Results
-Does the analysis presented match the analysis plan? NO**

R = The statistical analysis was conducted as initially designed for the research protocol, but a multivariate analysis was not performed due to the scarcity of data. We agree with the reviewer's suggestion to perform a more detailed analysis when the sample size is large enough for its application.

**-Are the results clearly and completely presented? NO.**

R = We tried to improve the presentation of the results for better clarity in the new manuscript.

**Results from matched case-control studies must be presented as discordant/concordant matched sets.**

R = The presentation of the results suggested by the reviewer is a good alternative, although the implementation is not possible due to the low amount of available data.

**-Are the figures (Tables, Images) of sufficient quality for clarity? NO. See above response regarding data presented in Tables. Figure 1 is unreadable. According to Results, it seems to be an epidemiological curve of arbovirus infection and GBS cases. Unfortunately, the figure is completely irrelevant for the purpose of the study.**

R = We agree that some of the tables are not clear, so they were modified to improve their understanding. Also, we agree that Figure 1 is irrelevant, for which it was deleted in the new version of the manuscript.

--------------------
**Conclusions
-Are the conclusions supported by the data presented? NO**

R = We agree with the reviewer. We have identified interpretation errors in the results section that do not support our conclusions. Therefore, as we realized our results are modest, the conclusions have been changed in the new version of the manuscript.

**-Are the limitations of analysis clearly described? NO**

R = We agree. The limitations of the study have been analyzed further and described in more detail.

**-Do the authors discuss how these data can be helpful to advance our understanding of the topic under study? NO**

R = We agree with the reviewer since no other points of view that do not support the results of the present and other similar articles were discussed.

**-Is public health relevance addressed? SOMEHOW.**

R = Having considered the reviewer's opinions, the discussion was further elaborated, the limitations were developed, and the conclusions have been modified for more clarity.

--------------------
**Editorial and Data Presentation Modifications?
- There are some other important limitations of this paper that were not included in the Reviewer Attachment.**

**The authors engaged in selective citation. They only cite articles that support their beliefs. In at least one case, the cited article does not support the authors claim (GeurtsvanKessel CH, et al.).**

R = We included only studies that could demonstrate the association through appropriate design, such as case and controls. However, it was not mentioned in the discussion that GeurtsvanKessel CH, et al. (2018) and Anaya JM, et al. (2017) found no association. Therefore, this statement was added to the new version of the manuscript.

Other publications that disagree with ZIKV and GBS association were neither included. These omissions have been considered in the new manuscript.

**- As mentioned above, the epidemiological curve (Figure 1) is irrelevant.**

R = We have deleted Figure 1 in the new version of the manuscript.

**- The authors mistakenly state that that an increase in GBS followed ZIKV outbreaks in Latin American countries. Previous studies have shown this was not the case. The claim that GBS increased in Mexico during 2016-2018 is unsupported by data. If that were the case, the increase could be easily explained by the implementation of GBS active surveillance, which started in 2015. At least one study (José Luis Soto-Hernández et al. doi: 10.3389/fneur.2019.00435) shows no increase in the incidence of GBS in 2016.**

R = The statements in lines 345-347 (345 Several reports indicate the probability of a causal association between ZIKV and 346 GBS due to the increase in the number of cases of GBS during ZIKV outbreaks 347 [18-20].) refer to what other authors have reported. However, as the reviewer correctly suggests, we do not think this is what happens. As the present research was not intended to measure whether there was an increase in GBS during the Zika outbreak in Mexico, no information on this regard was explored. Therefore, the statement in lines 445-451 has been deleted.

**- Some studies cited in support of a Zika-GBS association actually do not show such association.**

R = As we commented before, GeurtsvanKessel CH, et al. (2018) and Anaya JM, et al. (2017) found no association.

**- Needed references are missing through the paper.**

R = According to the suggestion, the present references were reevaluated, and new references, which we considered necessary, were included.

**- In several statements, authors confound temporal concurrence (coincidence) with statistical association.**

R = This reviewer's objection was not specific, for which it was not possible to address.

**- Even if ZIKVI caused GBS, the management of a patient with GBS should be based on his/her clinical characteristics, not on the risk factors that may have led to the development of the disease.**

R = We agree with the reviewer. GBS treatment is independent of its etiology. We included this information in the present manuscript, considering that it could be useful to readers.

**- Researchers do not need to give consent to participate as investigators.**

R = We agree. It was clarified in the limitations section of the manuscript. The invitation for researchers to participate in the study was made through the Epidemiological Surveillance Coordination, which is the administrative structure of the Mexican Social Security Institute (IMSS) that governs the activities of all the epidemiologists of the institution nationwide. As the participation of the epidemiologists was not mandatory, only those who voluntarily decided to participate in the study were included.

--------------------
**Summary and General Comments**

**Reviewer comments
I have read with great interest Grijalva et al.’s manuscript. They recruited a sample of 97 cases of Guillain-Barré syndrome (GBS) and 184 age, gender, and residence-matched controls without GBS. They found that acute Zika virus infection (ZIKVI) and dengue virus infection (DVI) increased the risk of GBS by 16.45 times (95% CI: 2.03 – 133.56) and 4.15 times (95% CI: 0.74 − 23.1), respectively. When combining both exposures, the risk of GBS increased 6.35 times (95% CI: 1.99 – 20.28). They concluded that there is an evident association between these infections and the risk of GBS. On the other hand, they compared the severity of GBS in infected and non-infected cases and concluded that cases with ZIKVI, but not those with DVI, have a more severe clinical picture, and recommended that ZIKVI status should be taken into account when treating patients with GBS.**

**Grijalva et al.’s is the second largest in a small group of case-controls studies of the association between ZIKVI and GBS risk. In spite of the obvious efforts of the investigators in conducting this study, its findings contribute little to clarify the possible causal nature of an association between arbovirus infections and risk of GBS. Indeed, like previous studies based on surveillance systems, this study has fundamental flaws that invalidate its findings. These flaws are described below.**

**Major Flaws
Detection bias occurs when the exposure of interest –ZIKVI or DVI− inﬂuences the diagnosis of the outcome −GBS.**

R = This is a misinterpretation: The inclusion criterion was GBS diagnosis and subsequently sampling for laboratory tests to determine exposure to arboviruses.

**This type of bias is present in all studies of the association of GBS with ZIKVI/ and DVI that are based on GBS cases recruited from surveillance systems in Latin America.**

R = It is imperative to clarify that this study was not supported by the epidemiological surveillance system of the Institute but independently, by invitation to participate through the Coordination of Epidemiological Surveillance to all hospital units of the country. We recognize that it was a serious translation mistake on our part.

**No country in Latin America had GBS surveillance before March 2016, when WHO/PAHO recommended the implementation of Zika-associated GBS surveillance.(1). Unfortunately, without adequate evidence of a causal association, WHO/PAHO declared GBS was the most frequent neurological complication of ZIKVI and recommended looking for evidence of GBS in all ZIKVI cases and vice versa. These recommendations were adopted by the IMSS and the Mexican Ministry of Health.(2-4) In fact, according to the IMSS Guidelines for the management of ZIKVI,(4) this recommendation had been implemented in the IMSS –the source of GBS cases for Grijalva et al.’s study− at least since November, 2014. Some of the authors in Grijalva et al.’s paper co-authored the IMSS Guidelines. The recommendation about GBS surveillance was also incorporated in the national guidelines for surveillance for vaccine preventable diseases,(2) under acute flaccid paralysis. Although surveillance guidelines do not directly call for the search of DVI in cases of GBS and vice versa, this is unavoidable due to the difficulties in differentiating DVI and ZIKVI, from both the clinical and laboratory perspective.**

R = What the reviewer pointed out is appropriate when a study is based on an epidemiological surveillance system; however, as noted previously, the present study was **not** supported by this system.

**The search for ZIKVI and DVI in cases of GBS, and vice versa, recommended in surveillance guidelines, results in an overestimation of the frequency of the exposure –ZIKVI/DVI− in GBS cases selected from a surveillance system. In consequence, if ZIKVI and DVI were not associated to GBS incidence, they will appear to increase the risk if GBS cases were selected from the IMSS surveillance system. Correspondingly, if ZIKVI and DVI increased the risk of GBS, the increase in risk would be overestimated.**

R = We agree that the detection bias occurs when the exposure of interest—ZIKVI or DVI—inﬂuences the diagnosis of the outcome—GBS. However, we consider that this bias is not committed by the study design. The study design was cases and controls, that is, once the GBS case was detected, it was studied for arbovirosis.

**This is not just a rational argument, as it is supported by data. Indeed, the magnitude of detection bias in Grijalva et al.’s study can be estimated using the incidence of Zika virus infection in Mexico (82 per 100,000)3 and the observed prevalence of ZIKVI in their sample of cases (8 out of 97).(5). The smallest OR for ZIKVI-GBS that would have resulted in 8 out of 97 cases infected with Zika virus was 100.6, and the observed prevalence of ZIKVI among GBS cases was 11.4 time higher than expected.**

R = In this study, once the cases of GBS were diagnosed, the risk factor was investigated, i.e., arbovirus infection. Therefore, the incidence of Zika at the national level is not related to the results given that it was analyzed individually, not in a population-based way.

**Briefly, the causal effect of ZIKVI/DVI on the risk of GBS could not be identified –measured or estimated− in Grijalva et al.’s study, due to the presence of detection bias. The large observed ORs in this study could be easily explained by detection bias. Unfortunately, there is no way to overcome this limitation.**

R = Indeed, the risk exposure has biases, which are produced by the arbovirosis diagnostic methods: PCR and serology. However, the diagnostic methods selected from the beginning were developed in the same way for both cases and controls samples. Therefore, detection bias was distributed equally for all groups.

**Selection bias: A fundamental principle in the design of case control studies is that case and control selection must be independent of the exposure. This means that in Grijalva et al.’s study, GBS cases and controls must have been selected independently of ZIKVI and DVI.**

R = We agree with the reviewer's opinion. The selection of the cases was conducted as mentioned.

**As explained above, this principle was violated for the case group, which included cases of concurrent −coincidental− GBS and ZIKVI, instead of cases of just GBS.**

R = We disagree with this comment. Cases were detected in hospital units for flaccid paralysis with a presumptive GBS diagnosis. If the diagnosis of GBS was confirmed by a neurologist and met the Brighton criteria (1 to 3), the patient was invited to participate. Until the patient signed the informed consent, it was considered as a case, and all clinical information and questionnaires were obtained, and blood and urine samples were collected.

As it was probably not explained correctly, cases were recruited regardless of their exposure to Zika, Dengue, or Chikungunya, for which a laboratory sample was collected to study these exposures.

In the Methods section, lines 94 to 97, the following is specified: After the interviews, serum and urine samples were collected to determine the exposure to ZIKV, DENV, and CHIKV during the first seven days after the onset of the neurological picture.

**In addition, the principle was violated during the selection of the control group, because only patients without fever were eligible as controls. Excluding patients with fever from the control group results in an underestimation of the frequency of the exposure (ZIKVI/DVI) in this group, because fever is common in patients with these diseases. It is not surprising that Grijalva et al. found a strong effect of these infections in the risk of GBS, because their approach resulted in overestimation of the exposure (ZIKVI and DVI) in cases and underestimation in controls. If no association existed, Grijalva et al.’s study was bound to show a positive association. If a positive association existed, Grijalva et al.’s study was bound to overestimate that association.**

R = In this case, we disagree with the reviewer. The control group in case-control studies is intended to provide estimates of the relative size of the denominators of the incidence rates for the compared groups. Therefore, each case in a case-control study should have been eligible as control before the time of disease onset. We acknowledge that omitting 'fever' from our control group may have led to an overestimation of the positive association. However, Zika cases frequently present without fever, for which we think that omitting fever in the control group still results in an adequate comparison.

**Another limitation of this study is the lack of details on eligibility and selection of controls. The only information provided was that controls were matched on state of residence, gender, and age to the cases, in a 2:1 ratio. Without further information, it is impossible to judge whether controls were a representative sample of the population from which the cases came from. For instance, were controls residing in high altitude places and those with hemostatic disorders eligible? Were patients with diseases potentially associated with the exposure excluded from the control group? Which diseases were excluded? How and where were eligible controls identified? In those cases where more than two matched controls were eligible for a given case, how were the two controls selected? Authors state they selected a non-probabilistic sample of controls. Unfortunately, unless the approach for control selection is explained it would be impossible to judge if it resulted in selection bias.**

R = We agree on the lack of details on eligibility and selection of controls, which has been addressed in the new manuscript. However, we consider that some precisions should not appear in the manuscript:

- **were controls residing in high altitude places and those with hemostatic disorders eligible?**

R = The cases and their respective controls came from nine coastal states and two non-coastal states, one with a tropical climate (Morelos) and another with a semitropical climate (Nuevo León). During the period of the study, positive cases for the three arboviruses were detected in all the states. On the other hand, we did not know the hemostatic disorders in the populations from which cases and controls were recruited. Regardless, the risk could be possible for both cases and controls.

- **Were patients with diseases potentially associated with the exposure excluded from the control group? Which diseases were excluded?**

R = The only exclusion criterion for controls was the presence of fever. We did not consider specific diseases.

- **How and where were eligible controls identified?**

R = Controls were identified by nursing personnel hired to perform this task: from the lists of inpatients or patients for elective surgery of the same hospital, or healthy relatives of these cases, who met the selection criteria. Consequently, individuals were invited to participate in the study and were given informed consent instructions. Once they agreed to participate and signed the consent, they were included in the study to continue with the corresponding procedures.

- **In those cases where more than two matched controls were eligible for a given case, how were the two controls selected?**

R = Controls were selected from the same locality as the cases (lines 81 and 82); thus, exposed to the same risk of becoming infected with a circulating virus.

Only two controls were selected for each case in this study. However, unfortunately, ten cases only got a single control.

The controls were paired by age (± 5 years), gender, and place of residence. They were not paired by the state of residence due to the enormous territory, but by municipalities, mainly because of their lesser territory. Pairing by municipality aimed to consider the risk of acquiring the disease of the participants similar to the cases. Each control was included within seven days from the date of the GBS case was included.

Regarding the *place of residence*, we consider that the above needs a broader explanation: The Royal Spanish Academy dictionary defines a *municipality* as the "Local entity formed by the neighbors of certain territory to manage their common interests autonomously."

In Mexico, the municipality is the primary cell that constitutes the political organization of the nation. A municipality is, at the same time, a territorial division and a local administrative entity which comprises a delimited territory, and consists of one or more localities.

In Latin America, not every country designates the smallest administrative divisions as municipalities. In other countries, such as Peru, Paraguay, or Panama, they are known as districts; in Costa Rica, as cantons, and in Chile, as communes. In Buenos Aires, Argentina, they are called parties, and in the Capital District of Bogotá, in Colombia, localities. In English, the equivalent could be shire or county.

We consider that no additional information is needed regarding the selection of paired controls.

**Out of 1030 GBS cases, only 97 were included in this study. The only eligibility criterion for was having a 1-3 Brighton level of certainty. This corresponds to 90% of all cases being Brighton level 4, as compared to 1.1% in previous GBS series.5 This remarkable difference suggests the accuracy of the diagnosis of GBS at the IMSS was incredibly poor or that other undisclosed GBS eligibility criteria were applied in this study. Without knowing the eligibility criteria, no judgment on the validity of the findings could be made.**

R = The 1030 cases correspond to hospital discharge that IMSS has nationwide, based on a clinical neurological diagnosis. This fact was noted as a reference point. It is not known whether the diagnosis of these 1030 cases was supported by the Brighton criteria as it was not an objective of this work. The 97 cases were those from whom, in addition to the clinical neurological diagnosis according to Brighton's criteria, blood and urine samples were collected, which allowed determining the risk of Zika, Dengue, or chikungunya before the onset of GBS.

**In summary, the causal effect of ZIKVI/DVI on the risk of GBS could not be identified –measured or estimated− in Grijalva et al.’s study, due to the presence of uncorrectable selection bias. There is no way to overcome this limitation.**

R = We consider that our responses so far discard the selection bias identified by the reviewer.

**Information bias:
Symptoms prior to the development of GBS were collected by interview. If cases were aware of they had GBS, they may have been trying, consciously or unconsciously, to concoct a reason why they got such a rare, serious disease. In consequence, they may have been more likely to report more symptoms and more severe symptoms than controls. In a similar fashion, if interviewers were aware of case/control status of the patient and the hypothesis being tested, they may have probed more deeply for ZIKVI and DVI symptoms in cases than in controls. Authors should describe how data were collected, to enable readers to judge the quality of their study.**

R = We agree with the observation of the reviewer. We did not register symptoms caused by viral infections before the development of GBS. As previously mentioned, patients were recruited once GBS was diagnosed (lines 157-159: “After the interviews, serum and urine samples were collected to determine the exposure to ZIKV, DENV, and CHIKV during the first seven days after the onset of the neurological picture”). In this case, the bias could be the recall of symptoms from the last two months, because even before collecting the hematic samples, participants were asked about their previous symptomatology. Controls were recruited within 7 days of the inclusion of the case and asked the same questions as the cases.

Although patients and interviewers ignored if the recruited cases were at risk of prior infection with any of the arboviruses, the interviews to identify previous diseases or symptomatology—of which individuals might be unaware—could be considered as another limitation of the study, which has been already addressed in the new manuscript.

**The fact that tests for ZIKVI and DVI were conducted after the diagnosis of GBS –up to 14 days−, makes reverse causality bias possible. In other words, it is possible that GBS cases got ZIKVI or DVI after they developed GBS.  In those cases, ZIKVI and DVI would be irrelevant exposures, as they could not cause the GBS.
None of these likely sources of information bias could be remedy in this study.**

R = Although the chance of demonstrating reverse causality is remote, of course, there is the possibility of this bias in this study.

**Confounding bias: Variables that are risk factors for GBS and are associated to ZIKVI and DVI could partly or completely explain the observed association between these diseases. For instance, patients from low socioeconomic status are more exposed to mosquito bites. This increases their risk of developing ZIKVI and DVI.**

R = Mexico, as other Latin American countries, has a large proportion of population in a situation of poverty. During the 2016-2018 period, in which the study was developed, between 43.6 and 41.9% of the Mexican population (53,418,151–52,425,887 inhabitants) were considered to be in a situation of poverty (National Council for the Evaluation of Social Development Policy, CONEVAL, 5 August 2019), and only 21.9% was considered not poor and non-vulnerable. As a result, almost 78% of the population was considered to be in a situation of vulnerability and poverty, which could explain that the low socioeconomic state could not be considered a substantial origin of bias.

<https://www.coneval.org.mx/Medicion/MP/Documents/Pobreza_18/Pobreza_2018_CONEVAL.pdf>

**They are also more likely to experience diarrheal diseases and acute respiratory infections, which are known to increase the risk of GBS. In fact, in a series of 17 prospective GBS cases from Mexico(6) 75% were positive for Campylobacter jejuni. However, Grijalva et al.’s study does not account for these or other potential confounders. Indeed, based on data in Table 5, the odds ratios for acute diarrheal disease and acute respiratory infections were 14.00 and 3.40, respectively. Therefore, these two risk factors could partly or completely explain the observed association between ZIKVI/DVI and risk of GBS.**

R = We agree with the reviewer's observation. This study did not consider other causes of GBS, such as *Campylobacter jejuni* that causes acute diarrheal disease and which, in Mexico, may be related to more than 50% of GBS cases. Other respiratory viruses also associated with the presence of GBS were neither considered, which is another limitation of the study that will be included in the new manuscript.

**Adjusting for confounding variables in this study is not possible, due to the small number of observations in strata defined by exposure, outcome, and confounder (see below).

Model specification bias
A fundamental limitation of Grijalva et al.’s study is that the data analysis does not correspond to the study design. In other words, the analysis does not reflect the mechanism by which the data were generated. Matching in a case-control study introduces selection bias. A matched analysis must be conducted to correct for the selection bias resulting from matching. Although the authors provide insufficient information about their analytical approach, besides mentioning a couple of statistical tests, it is obvious they calculated ORs using simple 2×2 tables, as if the study were unmatched.**

R = From a methodological point of view, pairing is a method to ensure that controls and cases are comparable to essential factors related to any disease. There are many risk factors and diseases related to age and gender that the results of a study may be unconvincing unless controls and cases are comparable regarding these two variables. In this study, the pairing was used as a methodological but not statistical issue.

**It is not surprising the investigators conducted an unmatched analysis. Matched analyses are based on the concordance of matched sets. Concordant triples or pairs (in this case) are those matched sets where the case has the same value of the exposure as the controls. If the case and controls in a matched set are all exposed or all non-exposed, the matched set does not contribute to the estimation of the OR. Only discordant pairs or triples –those with at least one control with a different exposure value as the case− contribute to the estimation of the OR. By looking at Grijalva et al.’s Table 1, it is easy to see that only matched sets from Nuevo Leon and Veracruz included discordant pairs. Moreover, only one discordant matched set –from Veracruz−has a control with ZIKVI. In consequence, a matched analysis of this case-control study –the correct analysis, considering the design− would be impossible. A correct approach to analyze the data from this study would be using conditional logistic regression. The Stata code in the attached document reproduces the data in Table 1, for GBS and ZIKVI, and then runs a conditional logistic regression. As expected, Stata cannot find an estimate for the conditional OR, because there are too few data points.**

R = This answer has been addressed previously.

Although the analysis of the results suggested by the reviewer is a good alternative, its use is not feasible in this study due to the fact commented by himself about the scarcity of the available data.

**Calculating ORs from 2×2 tables –the approach used by the investigators− not only leads to selection bias –because it ignores the matching−, but also fails due to the rather small number of data points. For instance, the OR for ZIKVI, from Table 1, would be (8/89)÷(1/183) = 16.45 (95% CI: 2.13, 733.64). The extremely large OR, with the excessively wide conﬁdence intervals indicates sparse data bias, instead of a strong effect of ZIKVI on GBS risk.(7) Basically, the fact that there is only one control that was exposed makes the OR extremely large and unreliable. A way to put this in simple terms is that the amount of data they had was insufficient to figure out with any certainty what was going on. In the presence of sparse data, the OR grossly overestimate the true effect of the exposure on the outcome. Combining ZIKVI and DVI does not solve this problem. The average of two biased ORs (bottom of Table 2) is another biased OR. Moreover, combining two very different ORs (16.45 for ZIKVI and 4.15 for DVI) makes no mathematical, nor biological sense.**

R = As previously mentioned, the pairing was used in this study as a methodological and non-statistical issue.

From the statistical point of view, an extremely large OR with excessively broad confidence intervals tending to infinity are inadequate. The new statistical analysis of the results has considered this and other comments from the reviewers.

**The problem of sparse data bias is even more marked in the comparisons of clinical characteristics of GBS patients with and without ZIKVI (Tables 4 and 5). For instance, the claim that mortality in GBS cases with ZIKVI infection is higher than GBS cases without ZIKVI was based on only one death in cases with ZIKVI.
Using Stata, I was unable to replicate the confidence intervals for ORs reported by the authors.**

R = The clinical characteristics were preferentially analyzed with Fisher's exact test due to sparse data. As indicated by the reviewer, we agree that the variable mortality was not statistically significant (as indicated in the last line of table 4) neither this manuscript nor the new one. Therefore, the paragraph from lines 390 to 393 of the discussion will be deleted.

**Briefly, the approach to the data analysis used in this study is incorrect and results in an overestimate of the effect of ZIKVI and DVI on GBS risk. A correct analysis, based on a conditional likelihood, is impossible. Both problems are due to the scarcity of the data.**

R = The issue of data scarcity prevented a conditional logistic regression analysis, which would have been an excellent option to analyze the results with appropriate sample size. Therefore, we consider the univariate analysis, which is usually recommended before the multivariate analysis to know the existing associations, to be valid in the present case.

Finally, considering the OR estimator and despite the extent of the confidence intervals due to the scarcity of data, we consider the solidity that the estimator has is not invalidated.

**DESCRIPTION OF THE MAIN CHANGES**

**Abstract**

Methodology/Principal findings:

- Statistical analysis: modified
- Laboratory evidence of viral infections: added
- Neurological symptoms of Dengue: deleted

**Author summary**

- Neurological symptoms of Dengue: deleted

**Introduction**

- No changes

**Methods**

Study design and participants

- The study was based on the Institutional Epidemiological Surveillance: deleted.
- Controls: The information was reorganized in a paragraph and additional details were included.
- Information about interviews in cases and controls was increased
- Acute viral infection and laboratory evidence of viral infection definitions were explained in detail.
- Laboratory analysis: the sequence of the lab tests was es pecified.
- Statistical analysis: The paragraph was rephrased and additional information was included.

**Results**

- Figure 1 was deleted
- Table 2 reorganized to provide clearer information.
- The laboratory evidence of viral infection information was double-checked. Unnecessary information was deleted.
- Neurologic alteration: Because only ZIKV infection was demonstrated, clinical ZIKV information was preserved and DENV information was deleted. Table 3 was created to compare signs and symptoms of all GBS cases, ZIKV+ and ZIK- cases. A new table (Table 4) was included to compare some clinical features and outcomes among all GBS cases, ZIKV+ and ZIK- cases.
- Suspected case symptoms paragraph only includes information related with GBS ZIKV+ cases. Additional data were deleted. A new table (Table 5) about symptoms prior the development of GBS only includes information related with controls, all GBS, ZIKV+ and ZIKV- cases.

**Discussion**

- The fourth paragraph from this section was modified to mention other studies that did not demonstrate any kind of association with ZIKV infection.
- The paragraph about co-infection was reduced to focus on these results.
- An additional paragraph regarding methodology problems found in eight case-control studies was included to improve future research projects.
- Neurological behavior. We deleted information about mortality related to ZIKV infection.
- Suspected disease symptoms prior GBS development. We added results from a new publication (Gongora-Rivera F, et al., PLoS ONE. 2020;15(3): e0230132). DENV disease suspected symptoms were deleted
- We deleted unconfirmed information related to unpublished data of the increase of ZIKV cases during the present study.
- Several limitations of the study were included.
- Conclusions. We deleted information regarding DENV related symptomatology and ZIKV related mortality.
