# Supplementary material for: Zika and dengue but not chikungunya are associated with Guillain-Barré syndrome in Mexico: A case-control study
Source: PLoS Negl Trop Dis. 2020 Dec 17;14(12):e0008032. doi: 10.1371/journal.pntd.0008032 (PMC7775118; doi:10.1371/journal.pntd.0008032)
Supplement: Attachment — Submitted filename: Reviewers responses & description manuscript changes RV2.docx [file pntd.0008032.s006.docx]

**REVIEWER 1**

**Methods**

**I believe the authors have addressed all the points I brought up in the first round of reviews.**

R = Requires no response.

**Results**

**I believe the authors have addressed all the points I brought up in the first round of reviews.**

R = Requires no response.


**Conclusions**

**I believe the authors have addressed all the points I brought up in the first round of reviews.**

R = Requires no response.

**Editorial and Data Presentation Modifications?**

**I believe the authors have addressed all the points I brought up in the first round of reviews.**

R = Requires no response.


**Summary and General Comments

I believe the authors have addressed all the points I brought up in the first round of reviews. They have also been very gracious in their acceptance of edits and suggestions from co-reviewers. Overall, this is a contribution to the literature from bother geographical area increasing the plausibility of the association of flaviviruses and GBS.**

R = Requires no response.

**REVIEWER 2**

**Methods**

**Yes**

R = Requires no response.

**Results**

**Yes. The paper is much improved from the previous version.**

R = Requires no response.


**Conclusions

Need to modify the conclusions to get rid of causal language. Causality cannot be established with this study design.**

R = We agree with the reviewer’s suggestion and modified the conclusions.

**Editorial and Data Presentation Modifications**

**(No Response)**

R = Requires no response.


**Summary and General Comments**

The manuscript is much improved and much clearer than the first version. It contributes with findings from an understudied disease and population. It requires one more round of editing to make sure there are no errors (e.g. check statistical significance, direction of < and > signs, etc), remove causal language in discussion and rather focus on association, and rectify some grammatical erroes (some of suggested modifications were marked on the pdf).

R = We agree with the reviewer’s suggestions. We found some errors and corrected them (small precisions on statistical significance and direction of < and > signs). We also removed causal language in discussion and focused on association. We also availed ourselves of additional grammatical help to improve the final version of the article.

**REVIEWER 3**

**Methods
All my comments are in the attached document. My original qualifications have not changed.**

R = Requires no response.

**Results**
**All my comments are in the attached document. My original qualifications have not changed.**

R = Requires no response.

**Conclusions**

**All my comments are in the attached document. My original qualifications have not changed.**

R = Requires no response.

**Editorial and Data Presentation Modifications**
**All my comments are in the attached document. My original qualifications have not changed.**

R = Requires no response.

**Summary and General Comments**
**This study have major design, data, and analytical flaws that invalidate its findings. These flaws cannot be addressed through data analysis, because the exposure and outcome were forced to be correlated during the diagnosis of the outcome and the evaluation of the exposure. There is no adjustment for confounding factors and selection bias was introduced by excluding controls with fever. Other major flaw is that the study is matched and this introduces a selection bias that can only be addressed by using a matched analysis. Unfortunately, a matched analysis is impossible due to very small number of discordant set. The later problem, the lack of data, result in sparse data bias, i.e. extremely large and unreliable odds ratios that the authors wrongly misinterpret as associations.**

Response = We are extremely concerned: despite having provided a broad, detailed explanation to the reviewer regarding what he deemed to be shortcomings in various aspects of our work, it appears that we are unable to persuade him to improve his opinion regarding at least some of those aspects. Nevertheless, and even though he has not changed his opinion, we believe that the result of our work –even with some limitations–, offers a degree of value worth considering regarding an association between GBS and the Flaviviruses Zika and Dengue in Mexico.

**DESCRIPTION OF THE CHANGES**

**Introduction**

- Mortality percentage was corrected and focused in USA.

**Methods**

- No changes.

**Results**

- Some clarifications on statistical significance (Table 2 and related paragraph) and direction of < and > signs.

**Discussion**

- The third paragraph from this section was modified to mention that we were unable to demonstrate an association between GBS with ZIKV acute infection.
- Conclusion paragraph describes that we only demonstrate laboratory evidence of ZIKV infection associated with GBS as well as laboratory evidence of the association of GBS with both ZIKV and DENV (flavivirus) infection.

All these changes are highlighted in blue

**Other Changes**

We availed ourselves of additional grammatical help to improve the final version of the article. Changes are highlighted in yellow.
